# The association between chronic periodontitis and oral *Helicobacter pylori*: A meta-analysis

**DOI:** 10.1371/journal.pone.0225247

**Published:** 2019-12-11

**Authors:** Xiang Wei, Hua-Qiang Zhao, Chuan Ma, Ao-Bo Zhang, Hao Feng, Dong Zhang, Chao Liu

**Affiliations:** 1 Shandong Province Key Laboratory of Oral Tissue Regeneration, School of Stomatology, Shandong University, Jinan, Shandong Province, China; 2 Department of Oral and Maxillofacial Surgery, School of Stomatology, Shandong University, Jinan, Shandong Province, China; 3 School of Stomatology, West China Hospital of Sichuan University, Chengdu, China; 4 Department of Oral and Maxillofacial Surgery, Qilu Hospital of Shandong University, Jinan, China; 5 Institute of Stomatology, Shandong University, Jinan, China; 6 Key Laboratory of Otolaryngology, NHFPC (Shandong University), Jinan, China; University of the Pacific, UNITED STATES

## Abstract

**Background:**

Epidemiological studies have shown that gastrointestinal *Helicobacter pylori* (*H*. *pylori)* infection is the main cause of chronic gastritis, but the relation between oral *H*. *pylori* and chronic periodontitis (CP) remains uncertain. A meta-analysis of published papers was performed to elucidate the correlation between oral *H*. *pylori* and CP.

**Method:**

To perform this meta-analysis, we searched papers published from 2000 to 2018 on PubMed, OVID, Springer Link, Chinese National Knowledge Infrastructure (CNKI) and Chinese Biology Medicine search engines. Pooled odds ratios (ORs) and 95% confidence intervals (CIs) for the correlation between *H*. *pylori* and CP were estimated. Heterogeneity, publication bias and subgroup analyses were also conducted.

**Results:**

A total of 918 papers on oral *H*. *pylori* and CP were collected, and 11 papers were in accordance with the inclusion criteria. Oral *H*. *pylori* was suggested to be correlated with CP. The results indicated that a *H*. *pylori*-positive state significantly increased the risk of CP 3.42 times (OR = 3.42; 95% CI = 2.71–4.31). A diagnostic test using polymerase chain reaction (PCR) showed a higher prevalence of *H*. *pylori* (OR = 3.70; 95% CI = 2.66–5.14) than did that using the rapid urease test (RUT) (OR = 3.13; 95% CI = 2.26–4.34).

**Conclusions:**

This paper demonstrated that CP was potentially correlated with oral *H*. *pylori* in adults and that oral *H*. *pylori* may be a possible risk factor for CP.

## Introduction

Periodontal disease (PD) is an oral disease epidemic, associated with a high risk of tooth loss in adults, particularly in the elderly population[1, 2]. PD usually presents in tooth-supporting tissues, including the periodontal membrane, alveolar bone and cementum. As a global disease burden[3], PD shows a close relationship with 200 systemic diseases[4], such as pulmonary disease[5], cardiovascular disease[6], head and neck cancer[7], survival of dental implants[8], and diabetes[9]. Therefore, it is important to identify risk factors causing PD.

PD is multifactorial in aetiology, but evidence in the literature suggests that the levels of specific Gram-negative microorganisms in subgingival plaque biofilm play a significant role in the initiation and progression of PD, particularly CP[10]. CP accounts for 95% of PD caused by microorganism infection and aetiology with local irritation. Most CP patients are adults, and the prevalence rate dramatically increases after 35 years of age[11]. The microorganisms in dental plaques and their products such as hydrogen peroxide are risk factors for CP[12, 13]. Recent studies have revealed that several types of microorganisms are related to the pathogenesis and development of CP, and some of these microorganisms have been identified[14], while others have not. Researchers are currently trying to isolate the specific microorganisms in CP and to determine the specific bacterium inducing this condition[15].

*H*. *pylori* is a Gram-negative, microaerophilic, rod-shaped bacteria. *H*. *pylori* is considered one of the most common bacterial infections in the human stomach[16]. Initially, *H*. *pylori* was isolated from gastritis mucosal biopsies in patients who suffered from chronic gastritis and reported worldwide in 1983[17]. *H*. *pylori* that selectively colonizes the gastric epithelium is the main reason for chronic gastritis, with an infection rate of 80%-95% in active chronic gastritis[18]. Several studies have shown that the presence of *H*. *pylori* is strongly associated with chronic gastritis and duodenal ulcers, which also indicated that the microorganisms in dental plaque and saliva play a role as a potential reservoir for *H*. *pylori*[19–21]. Miyabayashi suggested that *H*. *pylori* in dental plaque is a high risk factor for recurrent gastric infection[22]. Oral microorganisms play an important role in the balance of the human microbial community and the maintenance of human health. The human oral cavity is colonized by a large number of different microorganisms. Therefore, an imbalance of the oral flora contributes to oral diseases and even systemic diseases. The existence of *H*. *pylori* in dental plaque, saliva, oral mucosa and other parts of the oral cavity raises the question as to whether this bacterium should be categorized as a member of the normal oral flora and whether it can cause CP.

Zaric[23] valued the effect of triple therapy combined with periodontal therapy vs. traditional triple therapy alone for gastric *H*. *pylori* eradication in patients with *H*. *pylori* in the dental biofilm. The results showed that periodontal treatment in combination with systemic therapy could be a promising method to improve therapeutic efficacy and decrease the rate of recurrence. Therefore, any association between *H*. *pylori* in the oral cavity and CP can be used as a guidance to eradicate *H*. *pylori* by supragingival scaling, subgingival scaling and regular flossing to prevent, control and manage both oral *H*. *pylori* and CP.

The aim of this meta-analysis is to systematically review the published statistics regarding the correlation between oral *H*. *pylori* and CP and to provide a reference to identify the key resident organisms in patients with CP, which may offer valuable insight into the aetiology of dental disease.

## Materials and methods

### Ethics approval and consent to participate

All analyses were based on previously published studies, thus no ethical approval and patient consent are required.

The analysis protocol was strictly performed following the PRISMA guidelines (S1 File)[24].

### Search strategy

A systematic search was performed on PubMed, OVID, Springer Link, Chinese National Knowledge Infrastructure (CNKI) and Chinese Biology Medicine search engines from 2000 to 2018. Articles written in English and Chinese were included. Combinations of the following terms were input into the search engine (1) aetiological terms: *Helicobacter pylori* and *H*. *pylori*; and (2) outcome terms: periodontal disease, PD, chronic periodontitis, CP. In addition, we also reviewed the references cited in the searched articles to look for other related studies.

### Selection criteria

Two independent reviewers (XW and CM) provided fair judgement of the manuscripts. To avoid bias, discrepancies were determined through discussion with another reviewer (HQZ) from a third party. To confirm the detailed data in some studies, a final confirmation was obtained by asking the authors to contribute any related information and statistics.

To eliminate the interference from other factors, studies that are consistent with the following criteria were included: (1) subjects aged between 20 to 70; (2) study samples≥20; (3) retrospective studies; (4) a clear description of oral and/or gastric *H*. *pylori* infection; (5) a clear description of patient conditions (no use of antimicrobial agents within 6 months prior to the study, no history of previous scaling and root planning or periodontal therapy in the last 6 months); and (6) a clear description of diagnostic measurements to detect the presence of *H*. *pylori* (polymerase chain reaction, PCR and/or Rapid urease test, RUT). Saliva was collected and tested within 5 minutes. Gingival crevice bacteria were collected from the near middle of the mandibular first molar tooth, along with gargle. In PCR, the detection of *H*. *pylori* was performed[25]. In the RUT, the samples were inoculated into the RUT gel. If the test gel colour changed from yellow to red within 20 min, up to a maximum of 60 min, then the sample was regarded as positive for *H*. *pylori*. (7) The diagnosis for periodontitis was made according to the diagnostic criteria in periodontology.

The studies that did not meet these inclusion criteria were excluded during the initial review.

### Data extraction and quality assessment

Two reviewers (XW and CM) extracted the information and statistics to complete the standard collection form. Another reviewer (HQZ) settled any bifurcation problem by consulting with the respective authors on original articles. For every inclusive article, the following data are shown in the table: first author name, publication year, country, total number of cases, patient gender, diagnostic method, positive cases and duration.

### Data synthesis and statistical analysis

ORs and 95% CIs were employed to evaluate the outcome[26]. Due to the low or high level of heterogeneity, a fixed-effects model (Mantel-Haenszel method) or random-effects model was employed for analysis. *I*^*2*^ statistics were calculated to estimate the value of heterogeneity. For the *I*^*2*^ values, 25%, 50% and 75% were considered as low, moderate, and high scores, respectively[27]. To identify the influence of the two different methods (PCR and RUT) to detect *H*. *pylori*, a subgroup analysis was performed to reveal the sensitivity and relationship between the two diagnostic methods. Funnel plot asymmetry was regarded as an assessment for potential publication bias[28]. Probability values <0.05 were defined as statistically significant. All data analyses were performed with Review Manager software, version 5.3.

## Results

### Applicable studies and study characteristics

**Fig 1:**
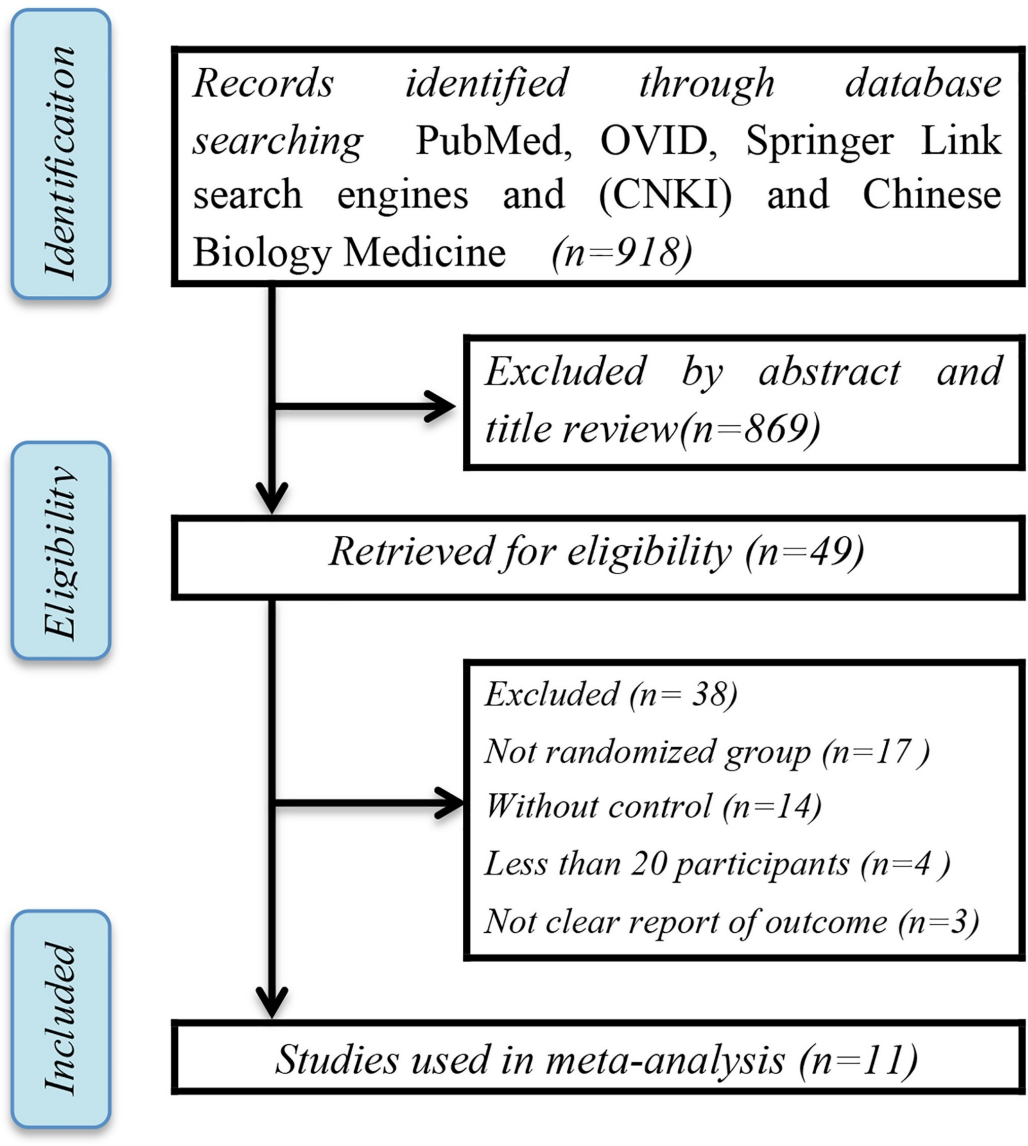
Flow chart of the study selection process.

Among 918 searched studies, 869 (94.7%) papers were excluded by abstract and title review. For the remaining 49 papers, 38 did not meet the criteria, and eventually, 11 papers[29–39] were chosen for this analysis (Fig 1). The characteristics of these studies are shown in Table 1. The analysis contained 1993 participants from 11 studies; in these cases, the gender of the participants was randomly selected. Among these individuals, 1319 had CP, while 674 were orally healthy and used as the control group. Among the 11 included studies, 6 studies used PCR as the diagnostic method to detect *H*. *pylori*, and remaining 5 studies utilized the RUT. Evaluation of the outcomes showed Hp-DNA and urease-positive results, respectively. However, not all studies showed the severity level of CP in detail.

**Table 1:** Characteristics of studies that used PCR and RUT to diagnose *H*. *pylori*.

*Study*	*Number of* *case group /Sex (M*:*F)*	*Number of* *control group /Sex (M*:*F)*	*Sample*	*Detection method*	*Detection index*	*Severity level* *(mild*:*moderate*:*severe)*	*H*.*pylori+/ case group (%)*	*H*.*pylori+/ control group (%)*	*Duration*
***Pei Zheng et al.2015/China[29]***	*70/43*:*27*	*70/44*:*26*	*Gingival crevice bacteria*	*PCR*	*Urease C gene&cagA gene*	*33*:*30*:*7*	*40/70(57%)*	*24/70(34%)*	*Jan 2013 to Dec 2014*
***Jing Yang et al.2015/China[30]***	*103/76*:*27*	*109/89*:*20*	*Subgingival dental plaque*	*PCR*	*Hp-DNA*	*NR*	*78/103(76%)*	*58/109(53%)*	*2012 to 2014*
***Souto et al.2008/Brazil[31]***	*169/NR*	*56/NR*	*Subgingival dental plaque*	*PCR*	*Hp-DNA*	*NR*	*85/169(50%)*	*6/56(11%)*	*NR*
***Al-Refai AN et al.2002/Saudi Arabia[32]***	*75/NR*	*60/NR*	*Gingival crevice bacteria*	*RUT*	*Urease*	*38*:*17*:*20*	*67/75(89%)*	*52/60(87%)*	*NR*
***Mohammed et al.2009/Saudi Arabia[33]***	*62/35*:*27*	*39/21*:*18*	*Subgingival plaque*	*RUT*	*Urease*	*NR*	*49/62(79%)*	*17/39(44%)*	*NR*
***MY Wang et al.2015/China[34]***	*120/53*:*67*	*80/37*:*43*	*Gingival crevice bacteria*	*RUT*	*Urease*	*NR*	*103/120(86%)*	*59/80(74%)*	*Sept 2012 to Mar 2013*
***LX Gong et al.2011/China[35]***	*496/262*:*234*	*66/35*:*31*	*Subgingival plaque*	*RUT*	*Urease*	*306*:*77*:*113*	*438/496(88%)*	*41/66(62%)*	*Jan 2010 to Oct 2010*
***Jing Li et al.2015/China[36]***	*85/55*:*30*	*91/69*:*22*	*Subgingival plaque*	*RUT*	*Urease*	*NR*	*69/85(81%)*	*52/91(57%)*	*Jun 2013 to Mar 2015*
***LP Wang et al.2001/China[37]***	*62/37*:*25*	*44/23*:*21*	*Gingival*	*PCR*	*Hp-DNA*	*16*:*29*:*17*	*21/62(34%)*	*4/44 (9%)*	*NR*
***Jing Gao et al.2011/China[38]***	*37/20*:*17*	*39/21*:*18*	*Subgingival plaque*	*PCR*	*Urease C gene&cagA gene*	*12*:*17*:*8*	*24/37 (65%)*	*15/39 (38%)*	*NR*
***YH Jiang et al.2002/China[39]***	*40/29*:*11*	*20/12*:*8*	*Subgingival plaque*	*PCR*	*Urease C gene&cagA gene*	*NR*	*29/40 (73%)*	*7/20 (35%)*	*NR*

NR: not reported

### Prevalence of *H*. *pylori* in periodontitis and non-periodontitis patients

**Fig 2:**
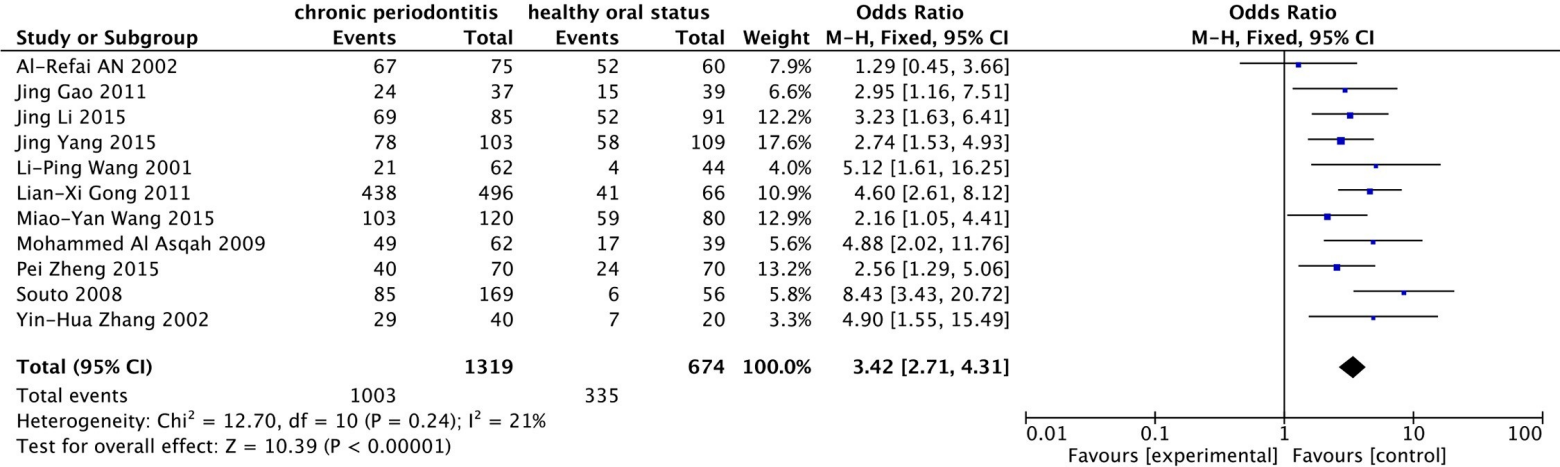
Presence of *H*. *pylori* in the case group and the control group.

**Fig 3:**
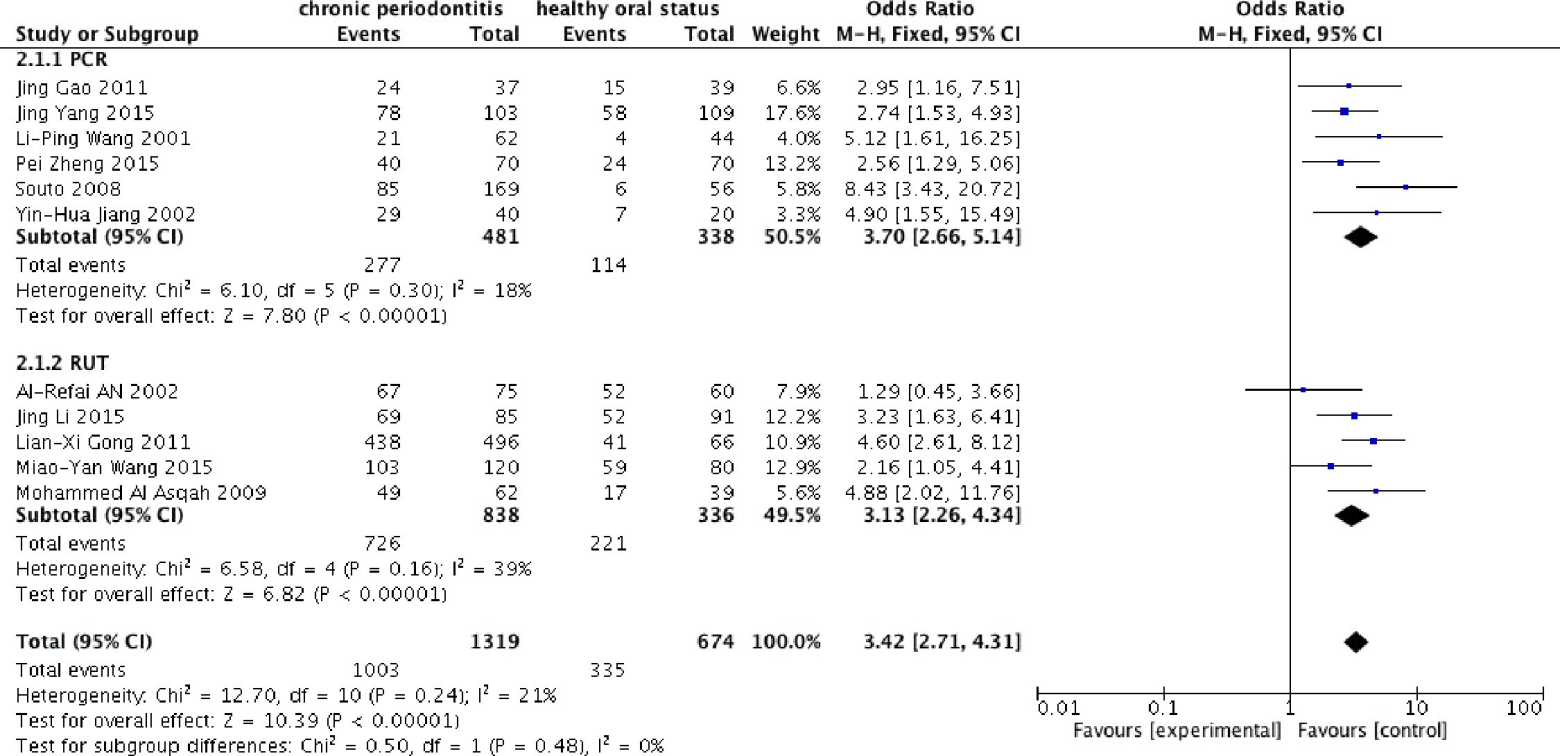
Stratified analysis of PCR and RUT.

The 11 studies[29–39] including 1993 individuals tested *H*. *pylori* by using two different diagnostic methods: PCR and RUT. As low heterogeneity was expressed between the studies (*I*^*2*^ = 21%), a fixed-effect model was applied. Compared with the *H*. *pylori*-negative population, *H*. *pylori*-positive patients had a significantly increased risk of CP (OR = 3.42, 95% CI = 2.71–4.31) (Fig 2). In the subgroup analysis of PCR and RUT, 6 studies utilized PCR techniques to detect *H*. *pylori* and 5 studies employed the RUT. To compare the outcomes of both techniques, a fixed-effect model was applied due to the relatively low heterogeneity. The frequency of *H*. *pylori* using PCR was higher than that determined with the RUT (Fig 3). The OR was 3.70, 95% CI = 2.66–5.14; *I*^*2*^ = 18% for PCR; in contrast, RUT showed an OR of 3.13, 95% CI = 2.26–4.34; *I*^*2*^ = 39%, which suggested that PCR had a higher detection sensitivity.

### Publication bias

**Fig 4:**
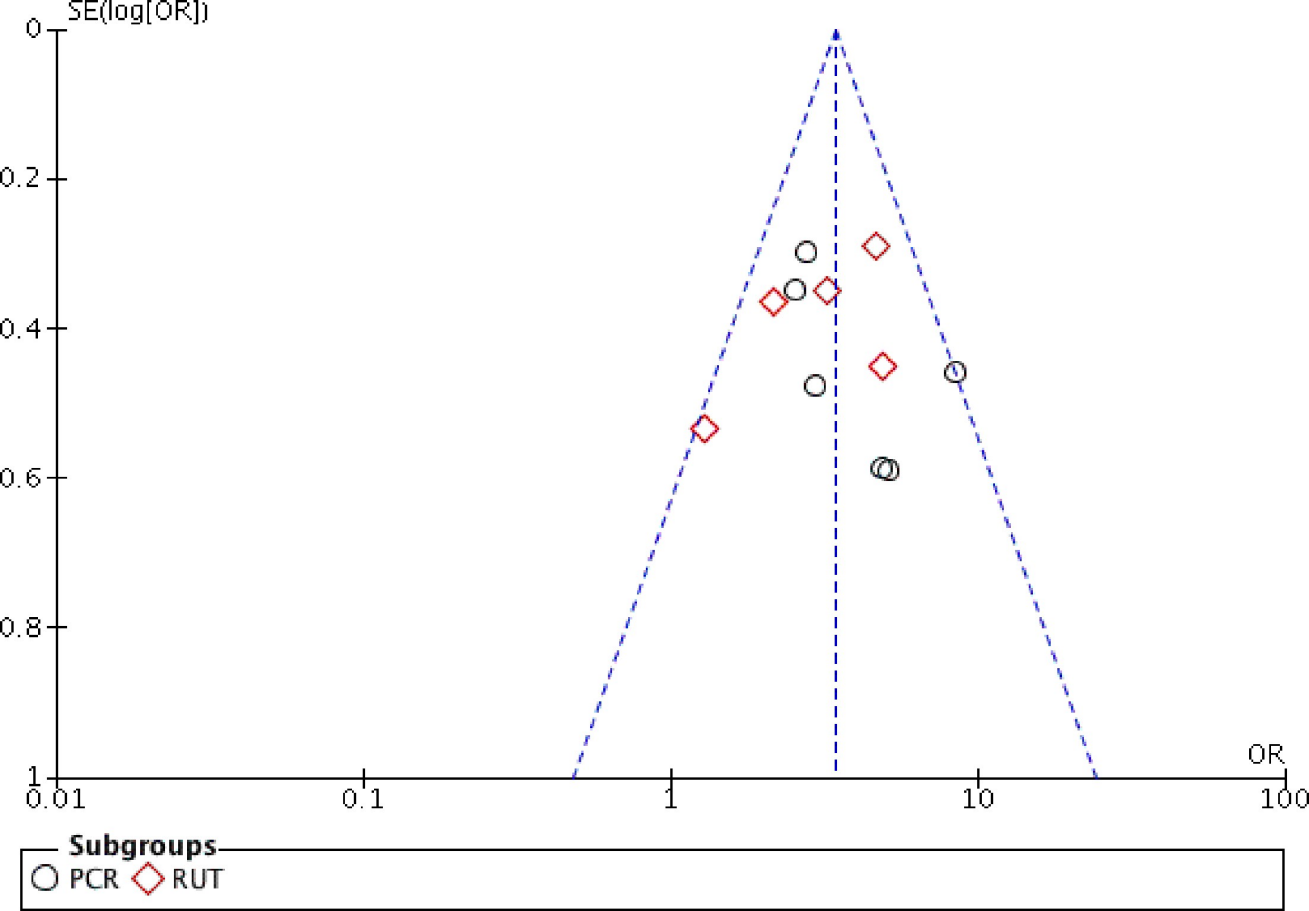
Funnel plot of PCR and RUT.

The funnel plot asymmetry revealed that there was no significant publication bias among studies on CP infections (Fig 4), despite the limited number of enrolments. Both PCR and RUT studies indicated relatively low heterogeneity, thus, publication bias was not substantial.

## Discussion

*H*. *pylori* is a common bacterium that causes gastrointestinal diseases and is considered a risk factor for many oral diseases[40], including CP. CP poses a great threat to the health of teeth as well as the health of an individual in general. The typical symptoms of CP include gingival inflammation, bleeding, periodontal pocket formation, alveolar bone resorption, alveolar bone height reduction, and tooth loss. The current preventive CP therapy is mainly focused on removing dental plaque. However, since it has been shown that dental plaque comprises a large number of commensal bacterium accompanied with a limited number of pathogenic bacterium, if oral *H*. *pylori* can be demonstrated to have a potential risk in causing CP, then targeted therapy may be a more effective way to prevent CP. In this meta-analysis, we found that the incidence of oral *H*. *pylori* in patients with periodontal diseases was significantly increased compared to that in the control group (3.42 times), while the negative correlations were no statistically significant. Although different methods for detecting the presence of *H*. *pylori* have been developed, the gold standard for the detection of *H*. *pylori* infection is still controversial. A recent study from India attempted to develop a “gold standard” to diagnose the *H*.*pylori* infection status[41]. PCR-based diagnosis may be regarded as a gold standard by designing primers for genes specific to *H*. *pylori*, such as urease operon genes, cag A and Hsp60. PCR provides an advantage of exploring the target DNA, regardless of the viability of the bacteria. A second benefit is that PCR is applicable for even a small number of target genes. RUT has also been considered as the standard in several studies owing to its high specificity, but the accuracy of the RUT is dependent on the size, number and bacterial density in the oral cavity.

Some studies that evaluated the effect of periodontal treatment on *H*. *pylori* showed a great reduction in *H*. *pylori* among patients who received periodontal therapy[42]. In periodontal therapy, the microbes colonized on the surface of the teeth is removed professionally by the dentist, along with other dental plaque control measures such as flossing and rinsing with mouthwash. This phase of treatment is considered to be of great importance because it is regarded as the etiotropic phase. In this period of time, the microbial aetiological factors of CP are eliminated. In previous studies, dental plaque showed some sort of resistance to systemically used antimicrobial agents because of biofilm properties[43, 44]. As a result, it is necessary to eradicate resident bacteria, including *H*. *pylori*, in a professional manner such as targeted therapy. Although dental biofilm cannot be eliminated completely, its pathogenicity can be lessened through an effective oral hygiene approach[45]. Thus, regular dental plaque removal is essential to prevent and control periodontal disease.

There are some limitations that should be considered to improve further investigations in the future. First, due to the lack of statistical data, the present study did not explore whether the severity of CP is changed when the counts of *H*. *pylori* are higher. Hence, relevant animal studies need to be conducted to determine whether there is a dose-response relationship between *H*. *pylori* and CP. Second, we were unable to explore whether there was a genetic background. There are many increased risk polymorphisms in CP, such as the interleukin-4 gene -590 C/T polymorphism[46] and the cyclooxygenase-2–1195G/A polymorphism[47]. Therefore, we suggest that polymorphisms should be studied in further research.

Another point of concern is that CP and oral *H*. *pylori* may be influenced by various risk factors, such as age, gender and social economic status[48]. In a large epidemiological study, Dye et al[49] found a remarkable association between the prevalence of *H*. *pylori* and advanced PD, even after adjusting for related socio-demographic factors. The gold standard detection methods for the presence of *H*. *pylori* are controversial[50]. The histology method is reliable but has never been accomplished with samples from the oral cavity. Although PCR is considered the gold standard, there are some defects, such as false positivity due to genetic sharing and false negativity due to low bacterium counts[41]. We also note that the amount of *H*. *pylori* that can be detected by PCR may be too small to cause any disease. Although different frequencies of *H*. *pylori* in dental plaques have been studied by multifarious investigators, the statistics collected from these studies have indicated that this microorganism can be found in dental plaque samples. The rate of oral *H*. *pylori* is associated with the incidence of CP, indicating a potential threat to increasing the depth of periodontal pockets and the pathological degree of periodontitis. The oral cavity functions as a gateway between the external environment and the gastrointestinal tract, and it plays a crucial role in assisting both food ingestion and digestion. Because *H*. *pylori* colonized the entrance of the digestive system, this bacterium is considered a high risk for gastric and duodenal ulcers and has been implicated in gastrointestinal infections. Therefore, gastric *H*. *pylori* infection patients suffering from CP are recommended to undergo initial periodontal treatment after systemic drug therapy, then the eradication rate of *H*. *pylori* will be increased. Additionally, the improvement of periodontal status has a strong effect on the elimination or survival of gastric *H*. *pylori*.

As for oral *H*. *pylori* control and the precautions for gastric and periodontal diseases, targeted therapy is necessary and essential to remove pathogenic bacteria.

## Conclusion

*H*. *pylori* infection is a common gastrointestinal infection that can cause pathological effects, increase oxidative stress and induce inflammatory responses. The interest in oral *H*. *pylori* has increased rapidly, since the presence of this bacterium in the mouth determines an oral-oral or oral-faecal method of transmission. Recently, *H*. *pylori* was frequently detected as the oral microorganism of subjects with periodontitis, suggesting that periodontal pocketing and inflammation may favour colonization by this kind of bacterium.

## Supporting information

S1 File PRISMA 2009 checklist. (DOC)

## Abbreviations

*H*. *pylori*: *Helicobacter pylori*
CNKI: Chinese National Knowledge Infrastructure
ORs: odds ratios
Cis: confidence intervals
PD: periodontal disease
CP: chronic periodontitis
PCR: polymerase chain reaction
RUT: rapid urease test
PI: plaque index
BI: bleeding index
